# 2013 Dengue Outbreaks in Singapore and Malaysia Caused by Different Viral Strains

**DOI:** 10.4269/ajtmh.14-0588

**Published:** 2015-06-03

**Authors:** Lee-Ching Ng, Yu-kie Chem, Carmen Koo, Rose Nani Binti Mudin, Faridah Mohd Amin, Kim-Sung Lee, Chong Chee Kheong

**Affiliations:** Environmental Health Institute, National Environment Agency, Singapore; Disease Control Division, Ministry of Health Malaysia, Putrajaya, Malaysia

## Abstract

Characterization of 14,079 circulating dengue viruses in a cross-border surveillance program, UNITEDengue, revealed that the 2013 outbreaks in Singapore and Malaysia were associated with replacement of predominant serotype. While the predominant virus in Singapore switched from DENV2 to DENV1, DENV2 became predominant in neighboring Malaysia. Dominance of DENV2 was most evident on the southern states where higher fatality rates were observed.

## Introduction

“United in Tackling Epidemic Dengue (UNITEDengue)” was launched on August 28, 2012, to initiate cross-border dengue case and virus surveillance to allow timely sensing of dengue situation in the region. Here we report the first significant outcome of UNITEDengue using data from Malaysia and Singapore and demonstrate the benefit of cross-border surveillance and data sharing, particularly for an endemic disease that does not receive attention for global or regional coordinated surveillance.

Dengue imposes a huge burden in the southeast Asia region, with frequent outbreaks.^1^ In urban Singapore, the economic impact of dengue from 2000 to 2009 was estimated to range between US$0.85 billion and US$1.15 billion.^2^ In Klang Valley, Malaysia, the impact in 2005 and 2009 was estimated to be US$38.2 million and US$56 million, respectively.^3^,^4^ In 2013, the two neighboring countries experienced unprecedented outbreaks. Malaysia reported a total of 43,348 cases, which was close to its peak in 2008 with 49,335 cases and more than twice the mean of 20279 cases in two previous lull years. Singapore reported a historical high of 22,170 cases, which was almost 50% more than the worst dengue epidemic in 2005 with 14,209 cases; and about four times the annual mean of 5308 cases in the 5 preceding lull years (2008–2012).

**Figure 1.**
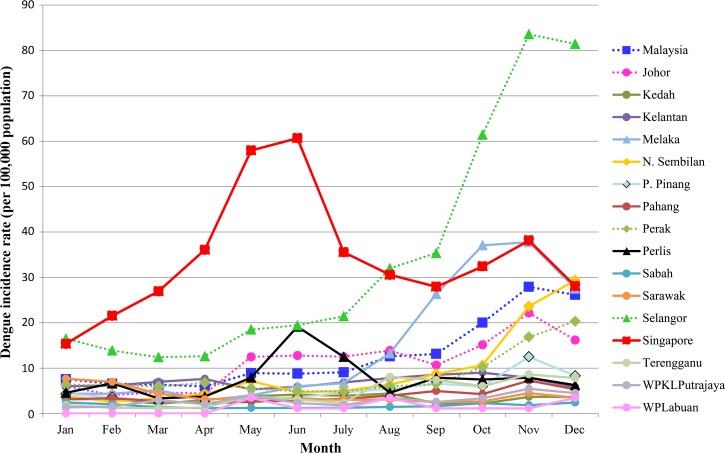
Dengue monthly incidence rate in Singapore and Malaysian States in 2013, case data extracted from UNITEDengue web portal.

Malaysia comprises West (Peninsular) Malaysia and East (Borneo) Malaysia, separated by the South China Sea. Singapore lies just south of the peninsula (Figure 1C) separated by the Straits of Johor but linked by a bridge and a causeway, each approximately 1-km long. About 182 million travelers and 61 million vehicles were reported to cross the border between the southern state of Johor and Singapore in 2011. This translates to an average of 167,123 vehicles and 498,630 travelers per day (Corporate Communications Division, Immigration and Checkpoints Authority). In addition, an average of 72 daily flights plies between Kuala Lumpur, the capital city of Malaysia, and Singapore. Both countries share the same tropical climate and many cultural similarities. Singapore, with a population of 5.4 million (2013), is a city with dengue endemic in most inhabited areas. On the other hand, Malaysia, with a population of 28 million, comprises both cities and rural villages.

## The Study

A shared secured web-portal with up-to-date database has been developed under UNITEDengue (www.unitedengue.org). Data shared include weekly incidence, monthly Dengue virus (DENV) serotype distribution and sequences of the envelope (*E*) gene of viruses of each state. The notification of dengue cases by clinicians is mandatory in Singapore and Malaysia While Singapore's official statistics are based on laboratory confirmed cases (NS1, PCR, or IgM tests), Malaysia's official case definition is based on clinical diagnosis according to the 1997 WHO guideline.^5^

Singapore's dengue virus surveillance system has previously been described.^6^ We have also previously reported that a switch in predominant circulating serotype could be an early warning of an impending dengue outbreak.^7^ Leveraging a Dengue serotype surveillance system that has been progressively set up since 2005, Malaysia set up an equivalent virus surveillance system in 2012 for monitoring and characterizing circulating dengue viruses in different states, using the same laboratory protocols. A total of 52 sentinel sites comprising hospitals and health clinics throughout the country were included in the surveillance system to capture both in- and outpatients (Supplemental Table 1). In each sentinel site, randomly selected clinically diagnosed dengue cases were tested for NS1 antigen. Virus serotype of each NS1 positive sample was determined by real-time PCR (RT-PCR) at the National Public Health laboratory. While the protocol aimed to have 5–10 NS1 samples from each sentinel site serotyped each week, some sentinel sites failed to consistently reach the number. Nevertheless, the compliance of sentinel sites in states with high disease burden (e.g., Selangor and Johor) suggested that the data are representative of the overall serotype pattern of the country. In 2012 and 2013, a total of 14,403 NS1 positive sera in Singapore (*N* = 9,512) and Malaysia (*N* = 4,891) were tested with RT-PCR to determine the DENV serotypes.^8^ Of them, 2,438 samples were successfully sequenced and analyzed for *E* gene as previously described.^6^

### Outbreaks associated with switch in predominant serotype.

**Figure 2.**
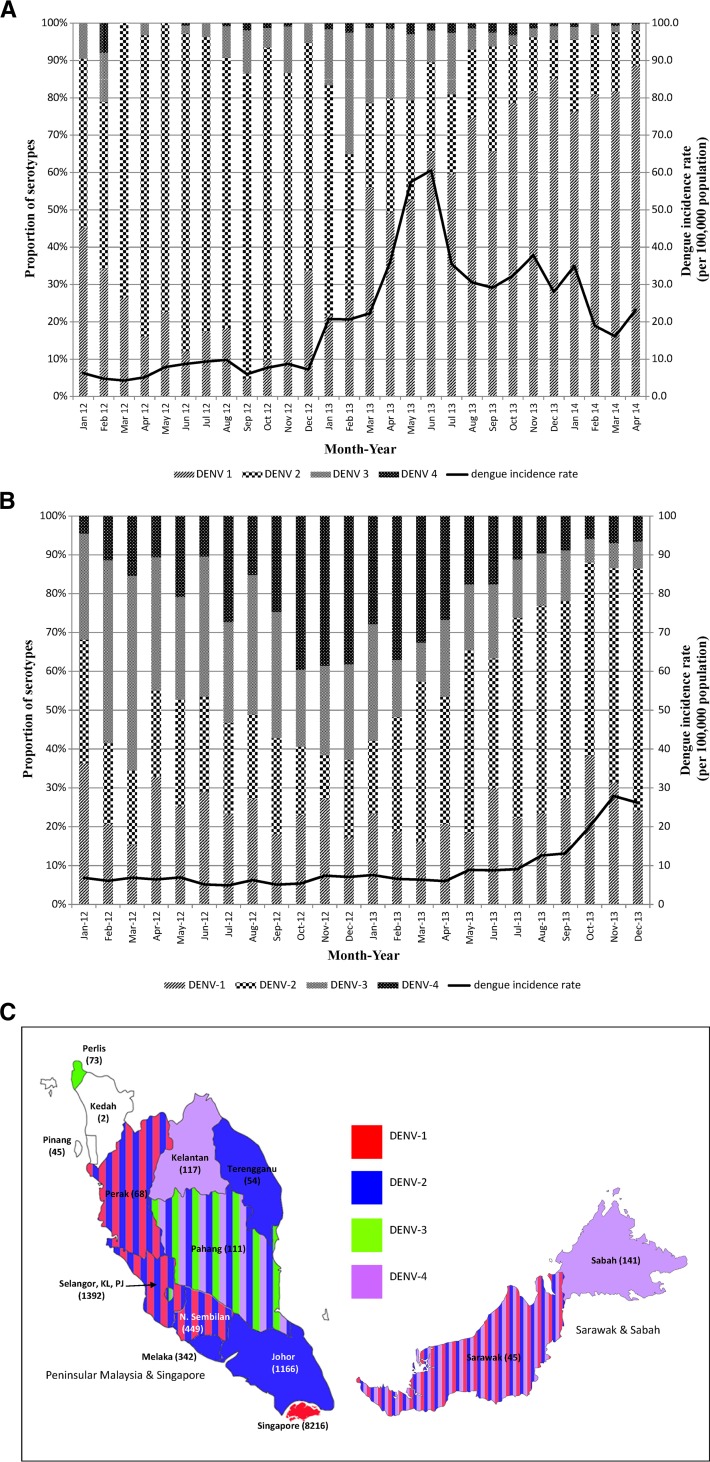
(**A**) Monthly distribution of DENV serotypes in Singapore 2012–2014. (**B**) Monthly distribution of DENV serotypes in Malaysia 2012–2013. Predominant serotype switched from DENV2 to DENV1 in Singapore, while it switched from DENV3/4 to DENV2 in Malaysia. (**C**) Spatial distribution of predominant DENV serotypes in Malaysia and Singapore (2013). Dominance of a single serotype by more than 50% is denoted by a single color. Stripes of different color denote almost equal proportion of more than one serotype. The number in each state denotes the number of samples used for serotype determination in 2013. N. Sembilan = Negeri Sembilan; KL = Kuala Lumpur; PJ = Putra Jaya.

In 2013, both Singapore and Malaysia experienced unprecedented outbreaks (Figure 2). Dengue incidence in Singapore peaked in June, reaching 815 cases a week. The number of cases continued to remain high till the end of the year—an unusual trend in Singapore. The incidence in Malaysia started to rise in July, which reached more than 2100 per week at the end of December. The number continued to remain above 2000 cases per week in the first two months of 2014 before subsiding in March 2014. Selangor reported the highest case burden of 55% of all cases in 2013, followed by Johor (11%). Interestingly, the incidence rate of Malacca state was higher than Johor in the fourth quarter of 2013. This suggests the population in Malacca was at higher risk than Johor during that period of time.

DENV serotype surveillance revealed that outbreaks in both countries were associated with a switch in predominant serotypes. The predominantly circulating virus in Singapore switched from DENV2 to DENV1 at the beginning of 2013. In Malaysia, DENV2 overtook a pre-existing DENV3 and DENV4 dominance pattern in 2013 (Figure 1A and B). DENV2 was most dominant in the southernmost states of Johor and Malacca (Figure 1C), where the proportion of DENV2 rose to 70–90% after August 2013. Incidentally, the fatality rates of 0.5% in those two states were unusually high when compared with that of the whole of Malaysia and Selangor (0.18% and 0.08%, respectively), where surveillance protocols were the same. The association of DENV2 dominance with high fatality is of concern although the reasons remain unclear. Hypothetically, it could be because of the inherent virulence of DENV2 strain or the antibody-dependent enhancement, which causes sequential infection of DENV1 (or DENV3) followed by DENV2 to be more severe. Such occurrences have been previously observed in Cuba^9^–^11^ and Brazil.^12^,^13^

### Envelope gene-based virus surveillance.

**Figure 3.**
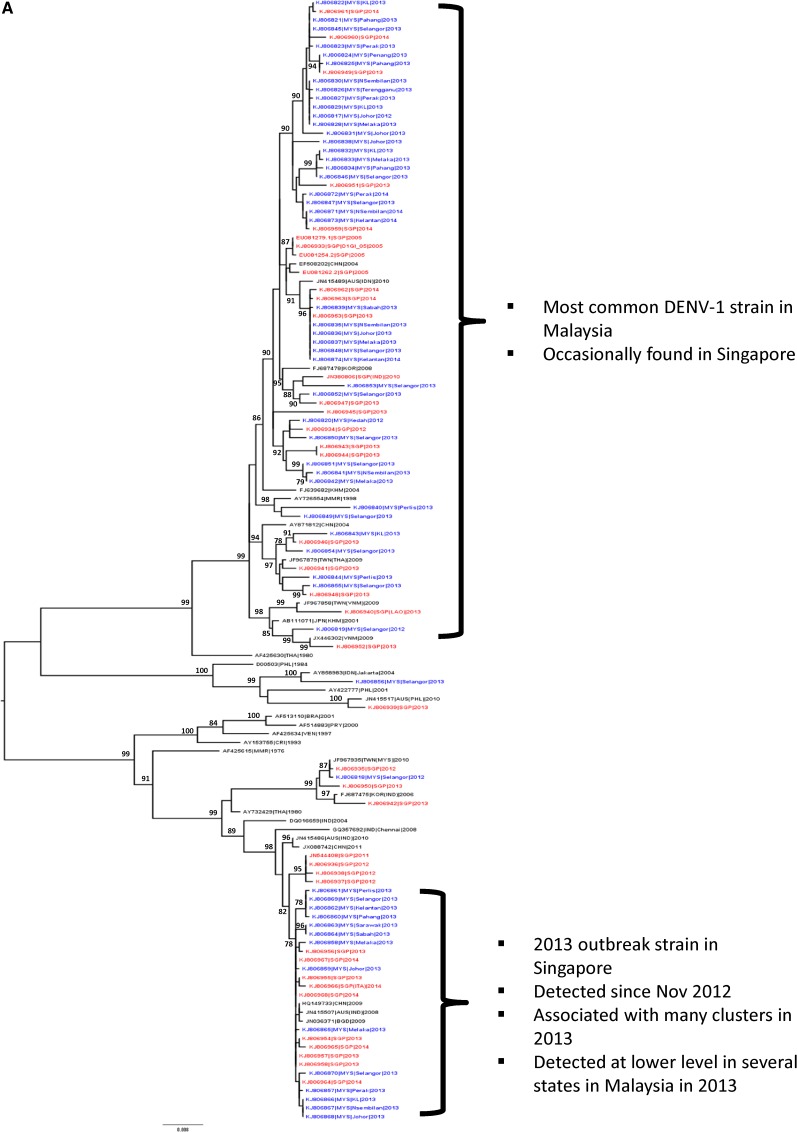

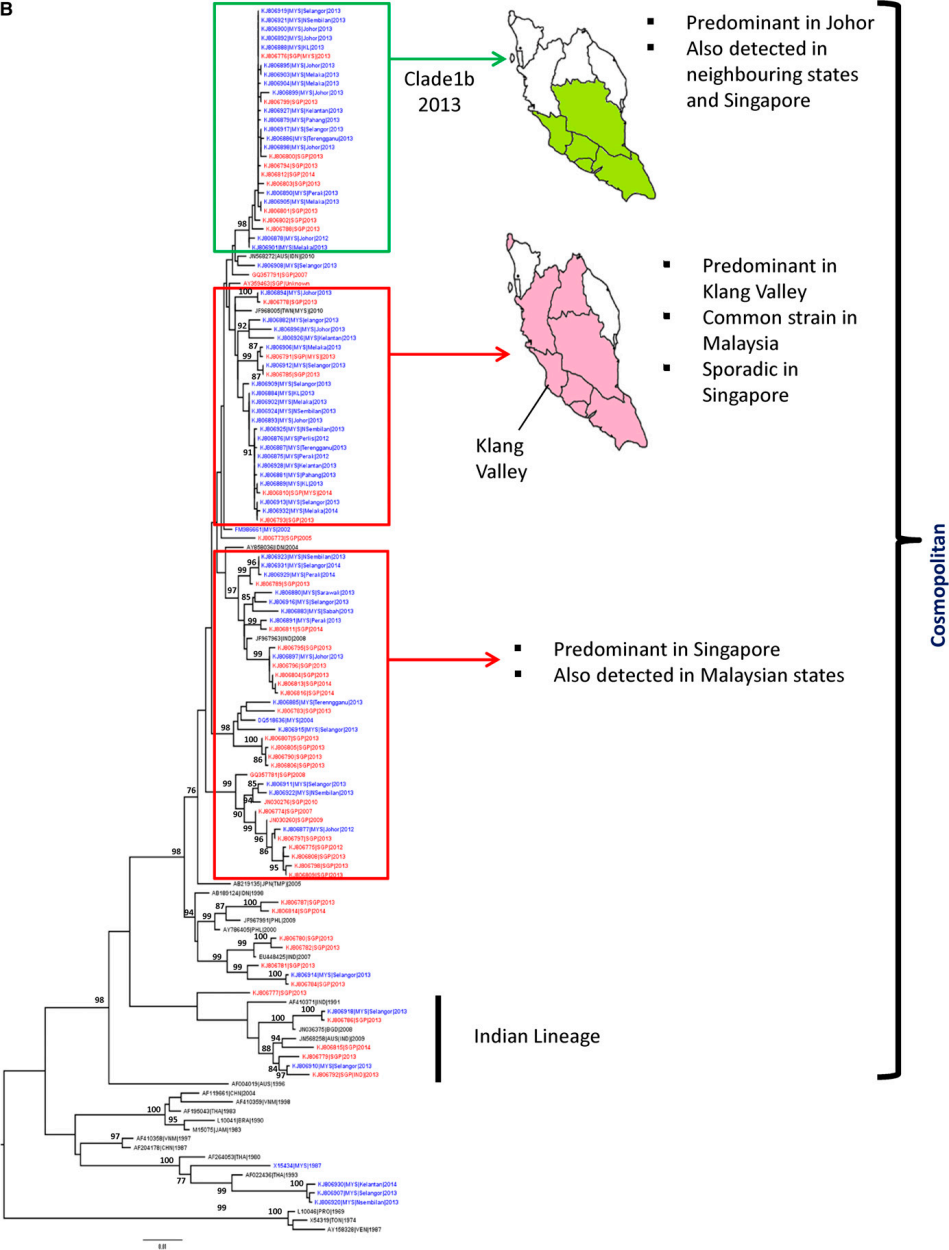
Phylogenetic relationships of global isolates of (**A**) DENV1 and (**B**) DENV2 based on the maximum likelihood method revealed the diversity of viruses in Singapore and Malaysia. They also highlight the DENV1 genotype III that caused the outbreak in Singapore, and the DENV2 clade1b that is predominant in Johor and Melaka (Malaysia) and appears to be associated with the high dengue fatality. Numbers on branches represent significant bootstrap percentages (> 75%). Due to the space constraints, the trees are based on representative viruses selected from 2,438 E gene sequences to highlight the significant clades of DENV1 and DENV2. Blue and red font represents Malaysian (MYS) and Singapore (SGP) sequences, respectively.

DENV1 strains circulating in Singapore during 2013 epidemic were first detected in November 2012 and belonged to genotype III (Figure 3A). From April 2013 onward, more than 50% of all viruses sampled in Singapore clustered in this clade (Figure 3A). Interestingly, only 42 (12%) of 349 DENV1 samples sequenced in Malaysia belonged to this clade (Figure 3A). A recent report by Teoh and others reported that DENV1 clade replacement is associated with recurrences of major DENV1 outbreaks in Malaysia and projected that Malaysia could experience another major outbreak because of in situ evolution of DENV1 genotype I.^14^ However, our data suggest that Malaysia should also be watchful of the DENV1 genotype III that contributed to a major outbreak in Singapore in 2013.

The predominant DENV2 strain found in Johor and Malacca belonged to the cosmopolitan genotype (named as cladeIb; Figure 3B) and was distinct from those that were predominant in the northern part of Malaysia. Though closely related to the cosmopolitan virus that caused Singapore's outbreak in 2007, and to strains that continue to circulate in Singapore,^6^ DENV-2 cladeIb in Johor and Malacca clustered distinctively (supported by a bootstrap value of 98%) from DENV-2 strains in Singapore (Figure 3B). Out of five dengue-related death cases in Johor, four were due to cladeIb viruses. Close monitoring in Singapore initially detected sporadic cases [40 (11%) out of 363 DENV2 sequenced] due to cladeIb, and seven of those 40 sequences were identical to the cladeIb strains in Johor. Incidentally, the first death case in Singapore in early 2014 was due to a virus from the same clade.

Genetic sequences of the *E* gene showed that there was a diversity of viruses in both countries and genetically identical viruses were shared between the two, suggesting frequent exchanges of DENV between Malaysia and Singapore. In general, the transmission pattern of dengue within a geographical region is influenced by the movement of infected persons within the region and the presence of the vector.^15^,^16^ Thus, neighboring countries are expected to exhibit similar dengue epidemiology and those with frequent inter-country movement of people could act as an epidemiological unit. However, predominant serotypes and strains driving the outbreaks in Singapore and Malaysia, and even in different states of Malaysia, were distinct. This suggests that DENV epidemiology in Singapore and Malaysia has distinguishable differences, contradicting a common belief that Malaysia and Singapore are of one epidemiology unit. This is not surprising due to very different immunologic backgrounds and entomologic environments in Malaysia and Singapore. Previous findings reported that different genotypes show a characteristic geographic distribution,^17^ implying a competitive advantage for individual virus strains in a particular region. Modeling work also suggests that the chances of an introduced DENV strain to survive and establish localized transmission in a particular location is dependent on the epidemiological landscape with a combination of factors such as density of mosquito population, fluctuation of biting frequency, and a period of cross-immunity.^18^ In addition, it has been suggested that the selection pressure on viral strains vary in different ecological environments and often acts on a local scale.^19^ As such, similar or identical viruses might not have equal chances to spread in different geographical locations. However, differences in dengue epidemiology between the two countries do not mean that they do not have substantial impacts on each other. A continuation of this cross-border program will reveal if a delayed impact will be observed. Nonetheless, the cross-border analysis of case and virus data could allow neighboring countries to better understand the dengue epidemiology in the region and warn each other of potential outbreaks proactively.

## Supplementary Material

Supplemental Table.
