# Supplementary material for: 2013 Dengue Outbreaks in Singapore and Malaysia Caused by Different Viral Strains
Source: Am J Trop Med Hyg. 2015 Jun 3;92(6):1150–5. doi: 10.4269/ajtmh.14-0588 (PMC4458818; doi:10.4269/ajtmh.14-0588)
Supplement: Supplementary file 1 [file SD3.pdf]

Supplemental Table 1

DENV serotype and genotype analysis in Malaysian states in 2013

No.	State	Sentinel site	Number of hospitals/clinics	Total number of samples received for dengue virology testing	Number of serotyped cases	Number of genotyped cases
1	Perlis	Hospitals	1	94	63	17
		Clinics	1	36	8	
		Non-Sentinel/outbreak*	N/A	25	2	
2	Kedah	Hospitals	1	13	1	1
		Clinics	2	11	0	
		Non-Sentinel/outbreak*	N/A	17	1	
3	Pulau Pinang	Hospitals	1	79	30	4
		Clinics	1	20	1	
		Non-Sentinel/outbreak*	N/A	74	14	
4	Perak	Hospitals	2	277	49	12
		Clinics	2	56	4	
		Non-Sentinel/outbreak*	N/A	171	15	
5	WPKL	Hospitals	1	226	192	66
		Clinics	1	21	7	
		Non-Sentinel/outbreak*	N/A	239	51	
6	Selangor	Hospitals	5	1510	1105	295
		Clinics	4	113	27	
		Non-Sentinel/outbreak*	N/A	56	10	
7	N. Sembilan	Hospitals	1	418	368	51
		Clinics	0	0	0	
		Non-Sentinel/outbreak*	N/A	144	81	
8	Melaka	Hospitals	1	452	167	64
		Clinics	1	138	34	
		Non-Sentinel/outbreak*	N/A	204	141	
9	Johor	Hospitals	4	1840	802	184
		Clinics	5	1368	329	
		Non-Sentinel/outbreak*	N/A	52	35	
10	Pahang	Hospitals	1	196	86	19
		Clinics	2	201	24	
		Non-Sentinel/outbreak*	N/A	12	1	
11	Terengganu	Hospitals	2	172	39	10
		Clinics	0	0	0	
		Non-Sentinel/outbreak*	N/A	97	15	
12	Kelantan	Hospitals	1	245	69	25
		Clinics	2	347	48	
		Non-Sentinel/outbreak*	N/A	1	0	
13	Sabah	Hospitals	1	159	111	12
		Clinics	1	145	29	
		Non-Sentinel/outbreak*	N/A	6	1	
14	Sarawak	Hospitals	6	108	30	9
		Clinics	2	24	7	
		Non-Sentinel/outbreak*	N/A	13	8	
		TOTAL	52	9380	4005	769

The table summarizes the DENV serotype and genotype analysis among NS1 positive sera collected in 2013 from both sentinel and non-sentinel/outbreak sites distributed across Malaysia. DENV = dengue virus.

*Ad hoc testing of NS1 positive sera collected from non-sentinel/outbreak sites. Each site was aimed to send at least two samples for laboratory testing and should not exceed 10% of the total cases per site.
